# Sero-prevalence and intrinsic factors associated with *Brucella* infection in food animals slaughtered at abattoirs in Abuja, Nigeria

**DOI:** 10.1186/s13104-017-2827-y

**Published:** 2017-10-10

**Authors:** Mabel Kamweli Aworh, Emmanuel Chukuwdi Okolocha, Emmanuel Jolaoluwa Awosanya, Folorunso Oludayo Fasina

**Affiliations:** 1 Department of Veterinary & Pest Control Services, Federal Ministry of Agriculture & Rural Development, FCDA Secretariat, Area 11, Garki, Abuja, Nigeria; 2 Nigeria Field Epidemiology and Laboratory Training Programme, Abuja, Nigeria; 3 Department of Veterinary Public Health and Preventive Medicine, Ahmadu Bello University, Zaria, Nigeria; 4 Department of Veterinary Public Health and Preventive Medicine, University of Ibadan, Ibadan, Nigeria; 5 Department of Production Animal Studies, Faculty of Veterinary Science, University of Pretoria, Onderstepoort, South Africa

**Keywords:** Brucellosis, Predisposing factors, Abattoir, Nigeria

## Abstract

**Background:**

Brucellosis, a neglected tropical food-borne zoonotic disease, has a negative impact on both animal and human health as well as tremendous socio-economic impact in developing countries where rural income relies largely on livestock breeding and dairy products. It is endemic in the animal population in Nigeria and is a recognized occupational hazard. This work was done to establish the sero-prevalence and predisposing factors of food animals in Abuja, Nigeria to *Brucella* infection.

**Results:**

Of 376 cattle, 203 sheep and 260 goats screened, 21 (5.6%); 19 (9.4%); 51 (19.6%) were positive, respectively for brucellosis with Rose Bengal Plate Test, and 2 (0.5%); 4 (2.0%); 10 (3.8%), respectively with c-ELISA. The likelihood of acquiring *Brucella* infection was higher among the Red Sokoto breed of goats compared to other breeds of goats (p = 0.05).

**Conclusion:**

This study showed that the prevalence of *Brucella* infection was low in food animals slaughtered at abattoirs in Abuja. However, of all animals screened, seropositivity to *Brucella* infection was highest in goats with Red Sokoto breed of goats more likely to acquire the disease when compared to other breeds.

## Background

Brucellosis also known as undulant fever is a neglected bacterial zoonosis of public health importance in Africa and certain parts of the world. This disease in animals has been eradicated in Australia, Canada, Europe, Israel, Japan and New Zealand [1]. It is also listed among the seven neglected zoonoses by the World Health Organization (WHO). Brucellosis has impacted greatly on both human and animal health with severe and tremendous socio-economic impacts, particularly in the developing countries where rural incomes depend heavily on livestock breeding and dairy products [2].

Of all the species of *Brucella: B. abortus, B. canis, B. melitensis* and *B. suis* are of public health importance. However, two species *B. melitensis and B. suis* have been reported to be more virulent in humans than *B. abortus and B. canis.* It is important to note that serious complications can occur with any of these species of *Brucella* [3].

Brucellosis, a foodborne zoonosis has caused considerable morbidity in humans in many parts of the world with major impacts on young children and elderly people [3]. People are at high risk of getting infected by drinking unpasteurized milk which is readily sold in parts of the country [4]. A recent study on brucellosis in the Federal Capital Territory (FCT), Abuja reported a sero-prevalence of 24.1% in abattoir workers [5].

In animals, it is endemic in most countries in Africa including Nigeria [6, 7] with a prevalence of 16.2% in slaughtered cattle population in sub-Saharan Africa [8] and 3.5% in Nigeria [9, 10]. The prevalence of the disease in slaughtered ruminants in Plateau State, Nigeria is higher and widespread (sheep, 14.5% and goats, 16.1%) [11]. Cadmus et al. have earlier reported a prevalence of 5.8% in cattle and 0.9% in goats in South Western Nigeria [12]. Previous studies have confirmed the problem of brucellosis in livestock with evidence of widespread infection in most parts of the country and attendant economic consequences [2, 10, 13–16]. Specifically, the serological prevalence obtained in different parts of Nigeria from different animal species ranged from 0.20 to 79.70% [12].

### Purpose of the study

Assessment of the prevalence of *Brucella* infection in food animals at abattoirs in some parts of Nigeria has been reported by very few researchers [17]. However in FCT, Abuja there is the paucity of data on the prevalence of *Brucella* infection and associated factors in food animals slaughtered at the abattoirs. The current study focuses on its intrinsic determinants in ruminants (cattle, sheep, and goats) slaughtered in two abattoirs in FCT, Abuja from June to August 2011 in order to provide indications about the extent of the problem in this study area. The main objective of this work was to determine the sero-prevalence and associated intrinsic factors with seropositivity to *Brucella* spp. in food animals (cattle, sheep, and goats) slaughtered at the abattoirs.

## Methods

### Study sites

**Fig. 1:**
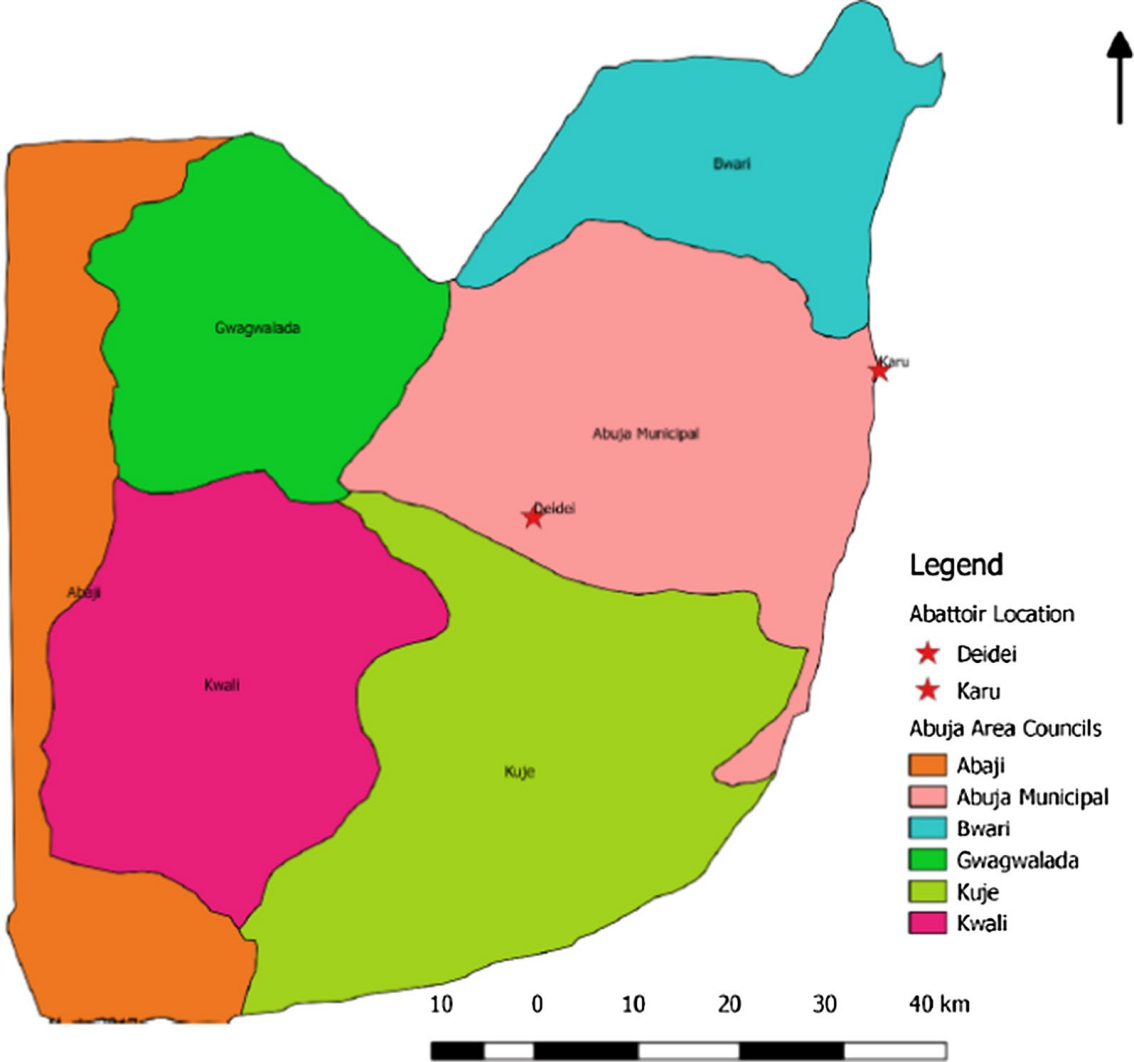
Map of Abuja showing the study area. This map highlights the six area councils in different colour codes namely: Abaji, Bwari, Kwali, Kuje, Gwagwalada and Abuja Municipal area councils. Our study was done at two abattoirs with high volume of daily slaughter of food animals (Karu, a government owned abattoir and Dei-Dei, a privately owned abattoir) located in the Abuja Municipal area council. (Source of the Map is the Federal Ministry of Agriculture and Rural Development, Abuja, Nigeria)

Two abattoirs with the highest population of food animals slaughtered on a daily basis were selected as study areas out of the five abattoirs across Abuja. This information was provided by the FCT Department of Agriculture and Rural Development. Karu abattoir (government owned) and Dei-Dei abattoir (privately-owned) were selected as study sites (Fig. 1).

### Study design

We conducted a cross-sectional study to determine the sero-prevalence of *B. abortus* and *B. melitensis* antibodies in sera of food animals slaughtered in the selected abattoirs. We also conducted an analytic cross-sectional study to establish the predisposing factors to *Brucella* infection in food animals.

### Study population

This comprised all cattle, sheep, and goats slaughtered at Karu abattoir and Dei-Dei abattoir at the time of the study.

### Sample size determination

The sample size was determined based on total population of food animals at the time of study with expected *Brucella* positive proportions of 32% in cattle [18], sheep (14.5%) and goats (16.1%) [11], q = 1 − p, 5% error margin and 95% confidence level (CI). Using ${\text{n = Z}}^{ 2} {\text{pq/d}}^{ 2}$, where p = 32% and q = 0.68 for cattle, 14.5% and 0.86 for sheep and 16.1% and 0.84 for goats, z = 1.96, d = 0.05, 10% non-response rate, the sample size was calculated and the following values were arrived at: cattle, n = 376; sheep, n = 203; goats, n = 260.

### Sampling method and data collection

Food animals slaughtered at these two abattoirs were included in the study. We used systematic random sampling with a sampling interval of four to select the food animals. We selected 18 cattle, 10 sheep and 13 goats per day until the sample size was obtained. Data on each species such as breed and sex were obtained through direct observation at the time of sample collection.

### Laboratory analysis

#### Sources of reagents

*Brucella abortus* (standard antigen, product code RAA0060) and *B. melitensis* (sensitive antigen, product code RAA2016) antigens for Rose Bengal Plate Test, sourced from Veterinary Laboratory Agency and OIE referral laboratory for Brucellosis, Weybridge, United Kingdom were used to screen cattle and sheep/goats respectively. Competitive Enzyme-linked Immunosorbent Assay (COMPELISA—400, product code RAI2006) kit also sourced from Veterinary Laboratory Agency, United Kingdom was used to screen all the animals.

#### Samples collection

Blood was collected aseptically from all food animals: cattle, sheep, and goats by a qualified veterinarian under strict hygienic conditions at the point of slaughter using sterile sample bottles. Five milliliter of blood from each animal was collected into a labeled, clean, sterile bottle and kept in slanted position on an ice-pack to clot for about an hour. Clear sera were separated from the clotted blood by decanting and further centrifuging at approximately 1000*g* for 10 min. A Pasteur pipette was used to apportion serum into labeled sterile sample bottles and stored at – 20 °C until needed for analysis.

### Serological testing of food animals

#### Rose Bengal Plate Test (RBPT)

We labeled each serum sample with a permanent marker and each was screened for *B. abortus* and *B. melitensis* antibodies using RBPT. The tests were done using the standard operating procedures provided in the OIE *Manual of Diagnostic Tests and Vaccines for Terrestrial Animals* [19]. The antigen and serum samples were brought to 22 ± 4 °C and 25–30 μl of each sample was placed on a white ceramic tile. The antigen bottle was gently shaken to ensure homogeneity and the same volume of antigen was mixed with each serum spot using a clean wooden rod for each test producing a circular zone. The mixture was gently agitated at room temperature on a rocker for about 4 min. It was read for agglutination immediately after the rocking was completed. Any visible agglutination reaction was considered to be positive. Agglutination reactions were interpreted as positive RBPT result for the affected animal.

#### Competitive enzyme linked immunosorbent assay (c-ELISA) method

The c-ELISA kit used was COMPELISA 400 for brucellosis diagnosis sourced from Veterinary Laboratory Agency, Surrey, United Kingdom. COMPELISA 400 which detects antibodies to smooth *Brucella* species was used to test the food animals (cattle, sheep, and goats) for *Brucella* antibodies. We constituted the reagents in the kit as directed by the manufacturers and performed the c-ELISA tests using the manufacturer’s protocol (Product code RAI2006). Test readings were taken at a wave length of 450 nm using a spectrophotometer Multiskan^®^ ELISA reader (Thermo Scientific, USA). A positive result was obtained when a sample showed optical density < 60% of the mean optical density of conjugate control wells [20].

### Statistical analysis

We entered, cleaned and analyzed data collected using Epi Info version 7 software. We determined proportions and compared the differences in sero-positive and sero-negative animals using Chi square tests and odds ratio (OR) for the investigated variables. We considered our test results as significant if the *p* value was < 0.05.

## Results

### Sero-prevalence of food animals

**Fig. 2:**
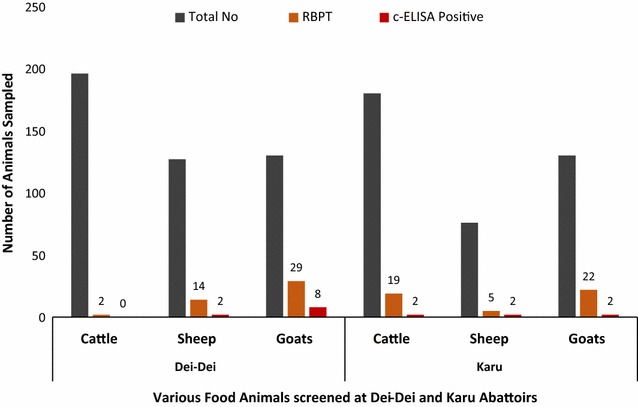
Distribution of seropositive animals among slaughtered food animals screened at Dei-Dei and Karu Abattoirs, FCT, Abuja, Nigeria. Cattle, sheep and goats slaughtered at Dei-Dei and Karu abattoirs in Abuja; were all screened with both RBPT and c-ELISA. Those positive for either of the tests were displayed along sides the total number of animals sampled. However more samples tested positive for brucellosis with RBPT when compared with c-ELISA

Sero-prevalence of food animals to *Brucella* infection varied from one abattoir to the other with the highest in cattle recorded at Karu abattoir. However, the highest sero-prevalence in sheep and goats was recorded at Dei-Dei abattoir. Overall 91 (10.8%) food animals were observed to be positive for brucellosis, among these 21 (2.5%) were cattle, 19 (2.3%) were sheep and 51 (6%) were goats (Table 1). Out of a total of 376 cattle screened at both abattoirs for *B. abortus*, 21 (5.6%) and two (0.5%) were positive for brucellosis by RBPT and c-ELISA, respectively. Of the 203 sheep screened for *B. melitensis*, 19 (9.4%) and four (2.0%) were positive for brucellosis by RBPT and c-ELISA respectively. Also out of a total of 260 goats screened for *B. melitensis*, 51 (19.6%) and ten (3.8%) were positive for brucellosis by RBPT and c-ELISA respectively. Among the food animals screened, the highest prevalence of *Brucella* infection was observed in goats both at Dei-Dei (22.3%) and Karu (16.9%) abattoirs in Abuja. The overall sero-prevalence in cattle, sheep, and goats was 5.6, 9.4 and 19.6% respectively (Fig. 2).

**Table 1:** Sero-prevalence of *Brucella* infection in food animals slaughtered in two abattoirs in FCT, Abuja, Nigeria

Abattoirs	Species	Total	RBPT positive	%	c-ELISA positive	%
Karu	Cattle	180	19	10.6	2	1.1
	Sheep	76	5	6.6	2	2.6
	Goats	130	22	16.9	2	1.5
Dei-Dei	Cattle	196	2	1.0	0	0
	Sheep	127	14	11.0	2	1.5
	Goats	130	29	22.3	8	6.2
Total food animals		839	91	10.8	16	1.9

### Risk factors associated with sero-prevalence in food animals

Factors associated with seropositivity to *Brucella* infection in the various food animals screened at Karu and Dei-Dei abattoirs in FCT are shown in Table 2. The only factor associated with *Brucella* infection in these food animals was the breed especially in goats (p = 0.05). The Red Sokoto breed of goats was more likely to acquire *Brucella* infection when compared to goats that were crossbred ([OR = 5.9; CI = 1.0–125.0] p = 0.05).

**Table 2:** Predisposing factors associated with *Brucella* seropositivity in food animals slaughtered at Karu and Dei-Dei abattoirs in FCT

Species	Characteristics	Sero-positive animals (%)	Sero-negative animals (%)	OR (95% CI)	p-value
Cattle	Breed				
	White Fulani	5 (3.6)	132 (96.4)	Ref.	
	Red Bororo	8 (5.7)	132 (94.3)	1.6 (0.5, 5.5)	0.42
	Sokoto Gudali	8 (8.1)	91 (91.9)	2.3 (0.7, 8.0)	0.14
	Sex				
	Male	20 (5.8)	326 (94.2)	1.8 (0.3, 38.5)	0.58
	Female	1 (3.3)	29 (96.7)	Ref.	
Sheep	Breed				
	Ouda	6 (9.8)	55 (90.2)	Ref.	
	Yankasa	13 (10.9)	106 (89.1)	1.1 (0.4, 3.4)	0.82
	Balami	0 (0.0)	23 (100.0)	0.0 (0.0, 1.7)	0.12
	Sex				
	Male	5 (8.5)	54 (91.5)	0.9 (0.3, 2.5)	0.78
	Female	14 (9.7)	130 (90.3)	Ref.	
Goats	Breed				
	Red Sokoto	50 (21.1)	187 (78.9)	5.9 (1.0, 125.0)	0.05*
	Cross breed	1 (4.3)	22 (95.7)	Ref.	
	Sex				
	Male	34 (17.6)	159 (82.4)	0.6 (0.3, 1.2)	0.17
	Female	17 (25.4)	50 (74.6)	Ref.	

* Values that are significant at p < 0.05

## Discussion

Our findings show that seropositivity to *Brucella* infection in food animals was highest among the goats when compared to cattle and sheep; this agrees with the results of Ahmed et al. where the seropositivity recorded in goats was 1.6 times higher than that of sheep [21]. Bertu et al. also reported a high sero-prevalence of 16.1% in goats in Plateau State, Nigeria [22]. Another recent study in Benue State, Nigeria by Ogugua et al. also supports our finding as the study reported a prevalence of 17.3% in goats [23]. The high seropositivity in goats may be due to the ability of the goats to shed *Brucella* organisms for long periods either in vaginal discharges or milk [24]. This has serious public health implications because of the zoonotic nature of the disease especially among abattoir workers who lack personal protective equipment, indulge in risky practices and are in close proximity with these food animals at the abattoirs [5].

This study showed that seropositivity to *Brucella* infection in cattle was low; consistent with findings of Cadmus et al. in Ibadan, Nigeria [25]. Our findings also revealed lower seropositivity to *Brucella* infection in sheep when compared to results from Bertu et al. in a sero-epidemiology survey of brucellosis in small ruminants which reported a higher sero-prevalence in sheep. Osman et al. in a similar study also reported a low prevalence of brucellosis in sheep which was consistent with findings from this study [26].

The only factor associated with *Brucella* infection in food animals observed in this study was that related to the breed of the animals especially in goats probably due to genetic variation which is an important factor in conferring resistance or tolerance to certain types to diseases [17]. This finding is in contrast to the reports of Mai et al. that brucellosis in animals is not breed specific [15]. However, findings from our study were consistent with that of Ogugua et al. who reported that breeds of goats were an important factor in determining sero-prevalence to *Brucella* infection as his study showed that high infection rates were observed among the Red Sokoto breeds of goats [23].

Predisposing factors reported by other studies for seropositivity to *Brucella* infection in cattle are breed and sex of animals [17]. Other studies reported that sex of the animals was a risk factor for seropositivity to *Brucella* infection [27, 28]. However, this study did not establish any association between sex of animals and seropositivity to *Brucella* infection probably because a low percentage of the food animal screened were seropositive to the *Brucella* organism. This finding is consistent with reports of Muma et al. in a study on risk factors for Brucellosis in ruminants reared in Zambia [29].

There appears to be a marked difference in the number of sero-positives detected by RBPT and c-ELISA in this study. This may be due to the stage of *Brucella* infection in the food animals as RBPT are usually more sensitive in detecting early *Brucella* infection than the c-ELISA test [30].

This study has limitations as we were unable to collect data on animal origin, age and gestational status of each animal slaughtered at the abattoir because the original animal owners were not present at the time of the study.

## Conclusion

The sero-prevalence of brucellosis among food animals slaughtered at abattoirs in Abuja was low. Of all the food animals screened, the highest seropositivity was recorded in goats. The breed of goats’ especially Red Sokoto breeds was an important factor associated with seropositivity for brucellosis in food animals. The following recommendations were made to the relevant authorities based on the results from our study: The need for regular surveys on brucellosis to be organized by Government at all levels among food animals slaughtered at the abattoirs as a lot of data could be generated for its control and eradication. The application of test-and-slaughter methods especially in the small ruminant population would further strengthen the control and eradication of brucellosis. Future work is also being recommended to focus on the origin of the goats slaughtered at these abattoirs so as to generate specific information to the government on caprine brucellosis outbreak hot-spots in the country.

## Abbreviations

B: *Brucella*
c-ELISA: competitive ELISA
FCT: Federal Capital Territory
OIE: World Organization for Animal Health
RBPT: Rose Bengal Plate Test
WHO: World Health Organization
